# Supplementary material for: Facilitators and barriers to the implementation of a caregiver-led training programme for caregivers of children with cerebral palsy in rural Malawi
Source: PLoS One. 2026 Sep 1;21(9):e0356240. doi: 10.1371/journal.pone.0356240 (PMC13533385; doi:10.1371/journal.pone.0356240)
Supplement: Supplementary file 2 [file pone.0356240.s003.docx]

**RESPONSE TO REVIEWERS**

| **Reviewer comment** | **Author response to reviewer** | **Reference**  **(According to manuscript file with tracked changes)** |
| --- | --- | --- |
| **Major issues** |  |  |
| 1. **Methods**   **Selection of study population (p7, lines 149-151)**  In such a small sample where purposive sampling is used, the potential for bias is high. More detail on what criteria were used, who selected participants, as well as how selection bias was minimised should be included. | Thank you for this important comment. We have revised the Study Participants section to provide additional detail on the sampling criteria, recruitment process, and steps taken to minimise selection bias.  Specifically, we clarified how participants were identified, who conducted the selection, and how variation across key characteristics was ensured | Page 7-8  Line 151-159 |
| 2. **Methods**   **Translation (p11, line 208)**  The use of Microsoft Word to translate transcripts is not recommended on its own for research purposes, as it does not capture nuance well. Nuance and tone would be important for phenomenological inquiry. The manuscript does not state how or to what extent this was done. This information would enhance credibility of findings. | Thank you for this important observation. We have revised the manuscript to clarify the transcription and translation process. Specifically, we have indicated that transcription and translation were conducted manually by bilingual members of the research team, with support from trained linguists experienced in translating the study tools. We have also described steps taken to preserve meaning and nuance, including cross-checking of transcripts and resolving discrepancies through discussion. These additions strengthen the credibility and rigour of the qualitative analysis. | Page 12-13  Lines 229-231 |
| 3. **Ethical considerations**   **Confidentiality (p12, line 224)**  Consent, assent and ethical approval were obtained, but information is missing regarding how data was managed to maintain confidentiality, or if this was done. This could affect participants’ responses in an interview and should be clarified. | Thank you for this important comment. We have revised the Ethical considerations section to include additional detail on how confidentiality was maintained. Specifically, we have described the use of participant codes, secure data storage, restricted access to study data, and anonymisation of transcripts and reports. These measures were implemented to protect participant identity and support open and honest responses during interviews. | Page 14  Line 258-263 |
| 4. **Conclusion (p24, line**   The conclusion should present phenomenological perspectives of the study participants concerning barriers and facilitators to the implementation of the programme. These are addressed well in lines 481 to 491. However, the concluding statement that, “the programme was effective” (Line 491) should perhaps be reworded so that it is clearly referring to implementation, not to the programme outcomes which would still need to be established in further study. Alternatively, the authors may be refer to the high acceptability and uptake of the programme in spite of contextual challenges, which was demonstrated in this study. As it stands, the comment may be misleading. | Thank you for this insightful comment. We agree that the original wording could be interpreted as implying programme effectiveness in terms of outcomes. We have revised the conclusion to more accurately reflect the phenomenological nature of the study by emphasising participants’ experiences of acceptability, relevance, and feasibility, rather than effectiveness. This ensures that the conclusions remain aligned with the study design and findings. | Page 27  Line 532-536 |
| **MINOR ISSUES** |  |  |
| 5. **Methods**   **Interviewer training (p8, lines p166-173)**  In order to ensure consistency in using the study guide, without influencing participants, it is useful to provide basic training or orientation, particularly where more than one interviewer is used. If this was performed, it would be useful to evaluate measurement bias. | Thank you for this helpful comment. We have revised the Methods section to clarify the interviewer preparation process, including orientation on the use of the semi-structured interview guide and procedures used to ensure consistency across multiple interviewers. We also clarified that all interviewers followed the same guide during data collection to minimise variability and potential bias. | Page 10  Line 186-189  Line 195-196 |
| 6. **Methods**   **Characteristics of the study population (p8, lines 154-160)**  Demographic information about the caregivers e.g., age, gender, education level, etc., and the proportions of children’s GMFCS level would have been useful to know. For example, factors such as family support requirements, ease of carrying out handling techniques, and transport needs would differ significantly between GMFCS levels. Knowing these proportions within the study group would help readers interpret their subject responses, especially in such a small sample. | we have included a new table (Table 2) presenting detailed demographic and clinical characteristics of the caregivers and children, including caregiver age, gender, education level, and distribution of children across GMFCS levels, to support interpretation of the findings | Table 2 |
| 7. **Study limitations (p24, lines 472-477)**   Study limitations are valuable for future research and could have been further elucidated. The sentence beginning with “Secondly,…” (line 474) does not make it clear how social desirability bias could affect responses adequately, because the relationship between the caregivers and the CBO is not sufficiently described elsewhere. Also, the potential for other forms of bias such as selection bias, is not mentioned. | Thank you for this helpful comment. We have expanded the Study limitations section to better explain the potential for social desirability bias by clarifying the nature of participants’ ongoing relationship with the implementing community-based organisation and how this may have influenced responses. We also added discussion of potential selection bias arising from purposive sampling and recruitment from programme participants. These additions strengthen the transparency and interpretability of the findings. | Page 26-27  Line 507-517 |
