# Supplementary material for: Facilitators and barriers to the implementation of a caregiver-led training programme for caregivers of children with cerebral palsy in rural Malawi
Source: PLoS One. 2026 Sep 1;21(9):e0356240. doi: 10.1371/journal.pone.0356240 (PMC13533385; doi:10.1371/journal.pone.0356240)
Supplement: Peer review history

# Peer review: Decision Letter 0

**DOI:** 10.1371/journal.pone.0356240.r001

**Authors:** Sascha Köpke

6 Apr 2026

Dear Dr. Bakuwa,

Thank you for submitting your manuscript to PLOS ONE. After careful consideration, we feel that it has merit but does not fully meet PLOS ONE’s publication criteria as it currently stands. Therefore, we invite you to submit a revised version of the manuscript that addresses the points raised during the review process.

Please submit your revised manuscript by May 21 2026 11:59PM. If you will need more time than this to complete your revisions, please reply to this message or contact the journal office at plosone@plos.org. When you're ready to submit your revision, log on to https://www.editorialmanager.com/pone/ and select the 'Submissions Needing Revision' folder to locate your manuscript file.

- A letter that responds to each point raised by the academic editor and reviewer(s). You should upload this letter as a separate file labeled 'Response to Reviewers'.
- A marked-up copy of your manuscript that highlights changes made to the original version. You should upload this as a separate file labeled 'Revised Manuscript with Track Changes'.
- An unmarked version of your revised paper without tracked changes. You should upload this as a separate file labeled 'Manuscript'.

If you would like to make changes to your financial disclosure, please include your updated statement in your cover letter. Guidelines for resubmitting your figure files are available below the reviewer comments at the end of this letter.

As the corresponding author, your ORCID iD is verified in the submission system and will appear in the published article. PLOS supports the use of ORCID, and we encourage all coauthors to register for an ORCID iD and use it as well. Please encourage your coauthors to verify their ORCID iD within the submission system before final acceptance, as unverified ORCID iDs will not appear in the published article. *Only*  the individual author can complete the verification step; PLOS staff *cannot*  verify ORCID iDs on behalf of authors.

We look forward to receiving your revised manuscript.

Kind regards,

Sascha Köpke

Academic Editor

PLOS One

**Journal Requirements:**

When submitting your revision, we need you to address these additional requirements.

1. Please ensure that your manuscript meets PLOS ONE's style requirements, including those for file naming. The PLOS ONE style templates can be found at

https://journals.plos.org/plosone/s/file?id=wjVg/PLOSOne_formatting_sample_main_body.pdf and

https://journals.plos.org/plosone/s/file?id=ba62/PLOSOne_formatting_sample_title_authors_affiliations.pdf

2. Please include a complete copy of PLOS’ questionnaire on inclusivity in global research in your revised manuscript. Our policy for research in this area aims to improve transparency in the reporting of research performed outside of researchers’ own country or community. The policy applies to researchers who have travelled to a different country to conduct research, research with Indigenous populations or their lands, and research on cultural artefacts. The questionnaire can also be requested at the journal’s discretion for any other submissions, even if these conditions are not met. Please find more information on the policy and a link to download a blank copy of the questionnaire here: https://journals.plos.org/plosone/s/best-practices-in-research-reporting. Please upload a completed version of your questionnaire as Supporting Information when you resubmit your manuscript.

3. We note that the grant information you provided in the ‘Funding Information’ and ‘Financial Disclosure’ sections do not match.

When you resubmit, please ensure that you provide the correct grant numbers for the awards you received for your study in the ‘Funding Information’ section.

4. Thank you for stating the following financial disclosure:

“TB is a fellow of the Consortium for Advanced Research Training in Africa (CARTA) which supported the research. CARTA is jointly led by the African Population and Health Research Centre and the University of the Witwatersrand and funded by the Carnegie Corporation of New York (Grant No—G-19-57145), Sida (Grant No:54100113), Uppsala Monitoring Centre and the DELTAS Africa Initiative (Grant No: 107768/Z/15/Z). The DELTAS Africa Initiative is an independent funding scheme of the African Academy of Sciences (AAS)’s Alliance for Accelerating Excellence in Science in Africa (AESA) and supported by the New Partnership for Africa’s Development Planning and Coordinating Agency (NEPAD Agency) with funding from the Wellcome Trust (UK) and the UK government. The statements made and views expressed are solely the responsibility of the Fellow.”

Please state what role the funders took in the study.  If the funders had no role, please state: "The funders had no role in study design, data collection and analysis, decision to publish, or preparation of the manuscript."

If this statement is not correct you must amend it as needed.

Please include this amended Role of Funder statement in your cover letter; we will change the online submission form on your behalf.

5. Please note that funding information should not appear in any section or other areas of your manuscript. We will only publish funding information present in the Funding Statement section of the online submission form. Please remove any funding-related text from the manuscript.

6. If the reviewer comments include a recommendation to cite specific previously published works, please review and evaluate these publications to determine whether they are relevant and should be cited. There is no requirement to cite these works unless the editor has indicated otherwise.

[Note: HTML markup is below. Please do not edit.]

Reviewers' comments:

Reviewer's Responses to Questions

**Comments to the Author**

1. Is the manuscript technically sound, and do the data support the conclusions?

Reviewer #1: Yes

**********

2. Has the statistical analysis been performed appropriately and rigorously?

Reviewer #1: N/A

**********

3. Have the authors made all data underlying the findings in their manuscript fully available?

The PLOS Data policy

Reviewer #1: Yes

**********

4. Is the manuscript presented in an intelligible fashion and written in standard English?

Reviewer #1: Yes

**********

Reviewer #1: Feedback to authors PONE-D-25-65705

Summary

This manuscript reports on a process evaluation that followed on a feasibility study of a 7-week caregiver-led programme designed to improve the knowledge and skills of caregivers of children with cerebral palsy (CP). Specifically, this study focused on the perspectives of trainers and trainees concerning the facilitators and barriers to implementation which were encountered. The report claims that the use of expert caregivers to train was integral to facilitate programme delivery, uptake and feelings of support by caregivers. The training was practical and well-organised. Programme-related barriers to implementation included the expert caregiver trainers not being coordinated and time constraints during more complex models. Most barriers came from external sources such as lack of social support, and transport.

The manuscript is well laid out, grammatically sound and has logical flow. Arguments and problem statements are presented concisely and clearly, and the need for the research is established. Methods are generally clear which would allow validation, although further clarity on a few aspects would improve transparency. The results section provides appropriate, clear tables and supplemental content that allows the abductive process to be observed. However, more demographic data could have been included about participants, allowing a more nuanced interpretation of statements and highlighted areas of potential bias that may have influenced responses. The discussion section presents a useful interpretation of the findings along with potential programme adapations; however, a richer analysis of the study limitations could be useful for future research. The conclusion accurately and succinctly summarises the barriers and facilitators of implementation of this programme but includes a statement about overall programme efficacy which is not easy to substantiate by the findings of this study.

Overall, this study provides a valuable contribution to the field of paediatric community health, especially in resource limited settings and the reviewer has no hesitation to recommend publication once the major issues have been addressed or adequately rebutted.

Major issues

Methods

1. Selection of study population (p7, lines 149-151)

In such a small sample where purposive sampling is used, the potential for bias is high. More detail on what criteria were used, who selected participants, as well as how selection bias was minimised should be included.

2. Translation (p11, line 208) The use of Microsoft Word to translate transcripts is not recommended on its own for research purposes, as it does not capture nuance well. Nuance and tone would be important for phenomenological inquiry. The manuscript does not state how or to what extent this was done. This information would enhance credibility of findings.

Ethical considerations

3. Confidentiality (p12, line 224)

Consent, assent and ethical approval were obtained, but information is missing regarding how data was managed to maintain confidentiality, or if this was done. This could affect participants’ responses in an interview and should be clarified.

Conclusion (p24, line

4. The conclusion should present phenomenological perspectives of the study participants concerning barriers and facilitators to the implementation of the programme. These are addressed well in lines 481 to 491. However, the concluding statement that, “the programme was effective” (Line 491) should perhaps be reworded so that it is clearly referring to implementation, not to the programme outcomes which would still need to be established in further study. Alternatively, the authors may be refer to the high acceptability and uptake of the programme in spite of contextual challenges, which was demonstrated in this study. As it stands, the comment may be misleading.

Minor issues

Methods

1. Interviewer training (p8, lines p166-173)

In order to ensure consistency in using the study guide, without influencing participants, it is useful to provide basic training or orientation, particularly where more than one interviewer is used. If this was performed, it would be useful to evaluate measurement bias.

2. Characteristics of the study population (p8, lines 154-160)

Demographic information about the caregivers e.g., age, gender, education level, etc., and the proportions of children’s GMFCS level would have been useful to know. For example, factors such as family support requirements, ease of carrying out handling techniques, and transport needs would differ significantly between GMFCS levels. Knowing these proportions within the study group would help readers interpret their subject responses, especially in such a small sample.

Discussion

3. Study limitations (p24, lines 472-477)

Study limitations are valuable for future research and could have been further elucidated. The sentence beginning with “Secondly,…” (line 474) does not make it clear how social desirability bias could affect responses adequately, because the relationship between the caregivers and the CBO is not sufficiently described elsewhere. Also, the potential for other forms of bias such as selection bias, is not mentioned.

Final comments

I commend the researchers on their groundbreaking work in paediatric community health that points to the importance of using what is readily available in resource-limited areas.

**********

what does this mean?). If published, this will include your full peer review and any attached files.

If you choose “no”, your identity will remain anonymous but your review may still be made public.

**Do you want your identity to be public for this peer review?**  For information about this choice, including consent withdrawal, please see our Privacy Policy

Reviewer #1: **Yes:** Shayne R van Aswegen

**********

[NOTE: If reviewer comments were submitted as an attachment file, they will be attached to this email and accessible via the submission site. Please log into your account, locate the manuscript record, and check for the action link "View Attachments". If this link does not appear, there are no attachment files.]

To ensure your figures meet our technical requirements, please review our figure guidelines: https://journals.plos.org/plosone/s/figures

You may also use PLOS’s free figure tool, NAAS, to help you prepare publication quality figures: https://journals.plos.org/plosone/s/figures#loc-tools-for-figure-preparation.

NAAS will assess whether your figures meet our technical requirements by comparing each figure against our figure specifications.

# Author comment: Author response to Decision Letter 1

**DOI:** 10.1371/journal.pone.0356240.r002

20 May 2026

EDITORIAL COMMENTS

1. Please ensure that your manuscript meets PLOS ONE's style requirements, including those for file naming. The PLOS ONE style templates can be found at

https://journals.plos.org/plosone/s/file?id=wjVg/PLOSOne_formatting_sample_main_body.pdf and

https://journals.plos.org/plosone/s/file?id=ba62/PLOSOne_formatting_sample_title_authors_affiliations.pdf

Response: I have formatted manuscript to meet Plos Ones requirements

2. Please include a complete copy of PLOS’ questionnaire on inclusivity in global research in your revised manuscript. Our policy for research in this area aims to improve transparency in the reporting of research performed outside of researchers’ own country or community. The policy applies to researchers who have travelled to a different country to conduct research, research with Indigenous populations or their lands, and research on cultural artefacts. The questionnaire can also be requested at the journal’s discretion for any other submissions, even if these conditions are not met. Please find more information on the policy and a link to download a blank copy of the questionnaire here: https://journals.plos.org/plosone/s/best-practices-in-research-reporting. Please upload a completed version of your questionnaire as Supporting Information when you resubmit your manuscript.

Response: I have completed the inclusivity questionnaire and added it as a supporting file

3. We note that the grant information you provided in the ‘Funding Information’ and ‘Financial Disclosure’ sections do not match.

When you resubmit, please ensure that you provide the correct grant numbers for the awards you received for your study in the ‘Funding Information’ section.

Response: I have updated the funding statement and provided correct grant numbers

4. Thank you for stating the following financial disclosure:

“TB is a fellow of the Consortium for Advanced Research Training in Africa (CARTA) which supported the research. CARTA is jointly led by the African Population and Health Research Centre and the University of the Witwatersrand and funded by the Carnegie Corporation of New York (Grant No—G-19-57145), Sida (Grant No:54100113), Uppsala Monitoring Centre and the DELTAS Africa Initiative (Grant No: 107768/Z/15/Z). The DELTAS Africa Initiative is an independent funding scheme of the African Academy of Sciences (AAS)’s Alliance for Accelerating Excellence in Science in Africa (AESA) and supported by the New Partnership for Africa’s Development Planning and Coordinating Agency (NEPAD Agency) with funding from the Wellcome Trust (UK) and the UK government. The statements made and views expressed are solely the responsibility of the Fellow.”

Please state what role the funders took in the study. If the funders had no role, please state: "The funders had no role in study design, data collection and analysis, decision to publish, or preparation of the manuscript."

If this statement is not correct you must amend it as needed.

Please include this amended Role of Funder statement in your cover letter; we will change the online submission form on your behalf.

Response: I have included the amended role of funder statement and included the revision in the cover letter

5. Please note that funding information should not appear in any section or other areas of your manuscript. We will only publish funding information present in the Funding Statement section of the online submission form. Please remove any funding-related text from the manuscript.

Response: I have removed funding information from the manuscript document

6. If the reviewer comments include a recommendation to cite specific previously published works, please review and evaluate these publications to determine whether they are relevant and should be cited. There is no requirement to cite these works unless the editor has indicated otherwise.

N/A

REVIEWER COMMENTS

Major Issues

1. Comment 1: Methods – Selection of study population (p7, lines 149–151)

In such a small sample where purposive sampling is used, the potential for bias is high. More detail on what criteria were used, who selected participants, as well as how selection bias was minimised should be included.

Response:

Thank you for this important comment. We have revised the Study Participants section to provide additional detail on the sampling criteria, recruitment process, and steps taken to minimise selection bias. Specifically, we clarified how participants were identified, who conducted the selection, and how variation across key participant characteristics was ensured.

Revision made: Page 7–8, Lines 151–159.

2. Comment 2: Methods – Translation (p11, line 208)

The use of Microsoft Word to translate transcripts is not recommended on its own for research purposes, as it does not capture nuance well. Nuance and tone would be important for phenomenological inquiry. The manuscript does not state how or to what extent this was done. This information would enhance credibility of findings.

Response:

Thank you for this important observation. We have revised the manuscript to clarify the transcription and translation process. Specifically, we indicated that transcription and translation were conducted manually by bilingual members of the research team, with support from trained linguists experienced in translating the study tools. We also described steps taken to preserve meaning and nuance, including cross-checking transcripts and resolving discrepancies through discussion. These additions strengthen the credibility and rigour of the qualitative analysis.

Revision made: Page 12–13, Lines 229–231.

3. Comment 3: Ethical considerations – Confidentiality (p12, line 224)

Consent, assent and ethical approval were obtained, but information is missing regarding how data was managed to maintain confidentiality, or if this was done. This could affect participants’ responses in an interview and should be clarified.

Response:

Thank you for this important comment. We have revised the Ethical Considerations section to include additional detail on how confidentiality was maintained. Specifically, we described the use of participant codes, secure data storage, restricted access to study data, and anonymisation of transcripts and reports. These measures were implemented to protect participant identity and support open and honest responses during interviews.

Revision made: Page 14, Lines 258–263.

4. Comment 4: Conclusion (p24)

The conclusion should present phenomenological perspectives of the study participants concerning barriers and facilitators to the implementation of the programme. These are addressed well in lines 481 to 491. However, the concluding statement that, “the programme was effective” (Line 491) should perhaps be reworded so that it is clearly referring to implementation, not to the programme outcomes which would still need to be established in further study. Alternatively, the authors may refer to the high acceptability and uptake of the programme in spite of contextual challenges, which was demonstrated in this study. As it stands, the comment may be misleading.

Response:

Thank you for this insightful comment. We agree that the original wording could be interpreted as implying programme effectiveness in terms of outcomes. We have revised the conclusion to more accurately reflect the phenomenological nature of the study by emphasising participants’ experiences of acceptability, relevance, and feasibility, rather than effectiveness. This ensures that the conclusions remain aligned with the study design and findings.

Revision made: Page 27, Lines 532–536.

Minor Issues

5. Comment 5: Methods – Interviewer training (p8, lines 166–173)

In order to ensure consistency in using the study guide, without influencing participants, it is useful to provide basic training or orientation, particularly where more than one interviewer is used. If this was performed, it would be useful to evaluate measurement bias.

Response:

Thank you for this helpful comment. We have revised the Methods section to clarify the interviewer preparation process, including orientation on the use of the semi-structured interview guide and procedures used to ensure consistency across multiple interviewers. We also clarified that all interviewers followed the same guide during data collection to minimise variability and potential bias.

Revision made: Page 10, Lines 186–189 and 195–196.

6. Comment 6: Methods – Characteristics of the study population (p8, lines 154–160)

Demographic information about the caregivers e.g., age, gender, education level, etc., and the proportions of children’s GMFCS level would have been useful to know. For example, factors such as family support requirements, ease of carrying out handling techniques, and transport needs would differ significantly between GMFCS levels. Knowing these proportions within the study group would help readers interpret their subject responses, especially in such a small sample.

Response:

Thank you for this valuable suggestion. We have included a new table (Table 2) presenting detailed demographic and clinical characteristics of the caregivers and children, including caregiver age, gender, education level, and distribution of children across GMFCS levels. These additions provide important contextual information to support interpretation of the findings.

Revision made: Table 2.

7. Comment 7: Study limitations (p24, lines 472–477)

Study limitations are valuable for future research and could have been further elucidated. The sentence beginning with “Secondly,…” (line 474) does not make it clear how social desirability bias could affect responses adequately, because the relationship between the caregivers and the CBO is not sufficiently described elsewhere. Also, the potential for other forms of bias such as selection bias, is not mentioned.

Response:

Thank you for this helpful comment. We have expanded the Study Limitations section to better explain the potential for social desirability bias by clarifying the nature of participants’ ongoing relationship with the implementing community-based organisation and how this may have influenced responses. We also added discussion of potential selection bias arising from purposive sampling and recruitment from programme participants. These additions strengthen the transparency and interpretability of the findings.

Revision made: Page 26–27, Lines 507–517.

Attachment Submitted filename: Response to reviewers.docx

# Peer review: Decision Letter 1

**DOI:** 10.1371/journal.pone.0356240.r003

**Authors:** Sascha Köpke

3 Aug 2026

Facilitators and barriers to the implementation of a caregiver-led training programme for caregivers of children with cerebral palsy in rural Malawi

PONE-D-25-65705R1

Dear Dr. Bakuwa,

We’re pleased to inform you that your manuscript has been judged scientifically suitable for publication and will be formally accepted for publication once it meets all outstanding technical requirements.

Within one week, you’ll receive an e-mail detailing the required amendments. When these have been addressed, you’ll receive a formal acceptance letter and your manuscript will be scheduled for publication.

An invoice will be generated when your article is formally accepted. Please note, if your institution has a publishing partnership with PLOS and your article meets the relevant criteria, all or part of your publication costs will be covered. Please make sure your user information is up-to-date by logging into Editorial Manager at Editorial Manager® and clicking the ‘Update My Information' link at the top of the page. For questions related to billing, please contact billing support.

If your institution or institutions have a press office, please notify them about your upcoming paper to help maximize its impact. If they’ll be preparing press materials, please inform our press team as soon as possible -- no later than 48 hours after receiving the formal acceptance. Your manuscript will remain under strict press embargo until 2 pm Eastern Time on the date of publication. For more information, please contact onepress@plos.org.

Kind regards,

Sascha Köpke

Academic Editor

PLOS One

Reviewers' comments:

Reviewer's Responses to Questions

**Comments to the Author**

Reviewer #1: All comments have been addressed

**********

2. Is the manuscript technically sound, and do the data support the conclusions?

Reviewer #1: (No Response)

**********

3. Has the statistical analysis been performed appropriately and rigorously?

Reviewer #1: (No Response)

**********

4. Have the authors made all data underlying the findings in their manuscript fully available?

The PLOS Data policy

Reviewer #1: (No Response)

**********

5. Is the manuscript presented in an intelligible fashion and written in standard English?

Reviewer #1: (No Response)

**********

Reviewer #1: (No Response)

**********

what does this mean?). If published, this will include your full peer review and any attached files.

If you choose “no”, your identity will remain anonymous but your review may still be made public.

**Do you want your identity to be public for this peer review?**  For information about this choice, including consent withdrawal, please see our Privacy Policy

Reviewer #1: **Yes:** Shayne R van Aswegen

**********

# Editor report: Acceptance letter

**DOI:** 10.1371/journal.pone.0356240.r004

**Authors:** Sascha Köpke

PONE-D-25-65705R1

PLOS One

Dear Dr. Bakuwa,

I'm pleased to inform you that your manuscript has been deemed suitable for publication in PLOS One. Congratulations! Your manuscript is now being handed over to our production team.

At this stage, our production department will prepare your paper for publication. This includes ensuring the following:

* All references, tables, and figures are properly cited

* All relevant supporting information is included in the manuscript submission,

* There are no issues that prevent the paper from being properly typeset

You will receive further instructions from the production team, including instructions on how to review your proof when it is ready. Please keep in mind that we are working through a large volume of accepted articles, so please give us a few days to review your paper and let you know the next and final steps.

Lastly, if your institution or institutions have a press office, please let them know about your upcoming paper now to help maximize its impact. If they'll be preparing press materials, please inform our press team within the next 48 hours. Your manuscript will remain under strict press embargo until 2 pm Eastern Time on the date of publication. For more information, please contact onepress@plos.org.

You will receive an invoice from PLOS for your publication fee after your manuscript has reached the completed accept phase. If you receive an email requesting payment before acceptance or for any other service, this may be a phishing scheme. Learn how to identify phishing emails and protect your accounts at https://explore.plos.org/phishing.

If we can help with anything else, please email us at customercare@plos.org.

Thank you for submitting your work to PLOS One and supporting open access.

Kind regards,

PLOS ONE Editorial Office Staff

on behalf of

Professor Sascha Köpke

Academic Editor

PLOS One
